# Determination of Commercial Animal and Vegetable Milks’ Lipid Profile and Its Correlation with Cell Viability and Antioxidant Activity on Human Intestinal Caco-2 Cells

**DOI:** 10.3390/molecules26185645

**Published:** 2021-09-17

**Authors:** Antonella Aresta, Stefania De Santis, Alessia Carocci, Alexia Barbarossa, Andrea Ragusa, Nicoletta De Vietro, Maria Lisa Clodoveo, Filomena Corbo, Carlo Zambonin

**Affiliations:** 1 Department of Chemistry, Aldo Moro University of Bari, 70126 Bari, Italy; antonellamaria.aresta@uniba.it (A.A.); nicoletta.devietro@uniba.it (N.D.V.); carlo.zambonin@uniba.it (C.Z.); 2 Department of Pharmacy-Pharmaceutical Science, Aldo Moro University of Bari, 70126 Bari, Italy; alexia.barbarossa@uniba.it (A.B.); filomena.corbo@uniba.it (F.C.); 3 Department of Biological and Environmental Sciences and Technologies, University of Salento, 73100 Lecce, Italy; andrea.ragusa@unisalento.it; 4 Department of Interdisciplinary Medicine, Aldo Moro University of Bari, 70126 Bari, Italy; marialisa.clodoveo@uniba.it

**Keywords:** animal and vegetable milks, milk lipid profile, free fatty acids, gas chromatography-mass spectrometry, Caco-2 cells, cell viability, oxidative stress

## Abstract

Lipids from milk are important nutritional components, although their health effects, especially for animal milks, are still questioned. Four types of commercial milks, two semi-skimmed animal milks (bovine and goat) and two vegetable ones (soy and rice), along with their total and free lipid fractions recovered by sequential centrifugation or by ethyl acetate extraction, respectively, have been analyzed. A higher antioxidant ability, reported as Trolox equivalent antioxidant capacity, was found for all raw milks compared to that of rice. This trend was confirmed, except for soy milk, as ROS reduction in Caco-2 cells. The free lipid fraction was shown to have the highest antioxidant potential in both chemical and biological tests. Moreover, goat and soy raw milks positively regulated Caco-2 cell viability after an inflammatory stimulus. This effect was lost when their total lipid fraction was tested. Finally, only the free lipid fraction from rice milk preserved the Caco-2 viability after LPS stimulation. Our data demonstrated that the lipid profile of each milk, characterized by GC-MS analysis, could contribute to dictate its biological effects, and, although additional in vitro and in vivo studies are needed, they could support the literature re-evaluating the health effects of animal-based versus plant-based milks in the intestinal cellular model.

## 1. Introduction

Animal and vegetable milks are emulsions in which lipids are structured into milk fat globules (MFGs) that contain non-polar lipids, mainly triacylglycerols (TAGs, 98%), but also other minor lipids, covered by a membrane containing amphipathic lipids and proteins [1,2]. The latter are composed of saturated fatty acids (SFAs) for around 70%, while the unsaturated FA (UFAs) could range from 1% to 3% with respect to the amount of total lipids [3]. Other minor components of milk fats are cholesterol and some plant sterols [4].

Ruminant milk fat contains more than 400 different fatty acids (FAs), and its composition differs among species and could be greatly influenced by animal nutrition [5]. Only 14 FAs represent more than 1% of the total lipid fraction in milk, and they are mainly SFAs, while most of them represent less than 0.01% of fats [6]. About 10% of SFAs in milk fat are short- and medium-chain FAs, while about 50% of SFAs are long-chain FAs, including palmitic (C16:0, 27%), myristic (C14:0, 10–12%), and stearic acid (C18:0, 9%). As for UFAs, about 25–30% are monounsaturated fatty acids (MUFAs), among which oleic acid (C18:1) is the most important, while 2–6% are polyunsaturated fatty acids (PUFAs), including ω-3 and ω-6 FAs, whose balance must be strictly regulated to induce a positive effect on human health [7,8,9,10,11,12].

Although FAs represent only a minor component of milk, they are an important nutritional component. Nevertheless, their effect on human health is a matter of debate [13]. Recent data showed that milk SFAs could modulate human health differently, e.g., short- and medium-chain SFAs did not have a negative impact on the blood lipid profile because they are easily digested and metabolized compared to long-chain ones [14]. On the other hand, SFAs support chronic inflammatory disorders leading to the onset and progression of multiple diseases, such as Inflammatory Bowel Disease and obesity [15,16]. Furthermore, milk fat intake is often associated with a higher risk of cardiovascular diseases due to its high content in long-chain SFAs, such as myristic and palmitic acids [17]. For this reason, the FAO and the WHO suggested that these SFAs, along with lauric acids (C12:0), raise the LDL serum concentrations. Therefore, they recommend that the human consumption of SFAs not exceed 10% of the total energy intake, and, when possible, SFAs should be replaced by ω-3 and ω-6 FAs to reduce the risk of arterial coronary diseases [18]. However, there is a growing consensus indicating that milk fat is a source of natural bioactive components with beneficial effects on human health, some of which are not typically found in significant amounts in our diet. For example, milk fat is almost the only source of butyric acid (C4:0), which plays an essential role in maintaining microbiota health, and it has been reported to inhibit intestinal inflammation and carcinogenesis, as well as to induce antimicrobial activity [19]. Positive effects have also been attributed to MUFAs and PUFAs, as well as to conjugated linoleic fatty acids (CLAs) [20]. Several clinical and epidemiological studies have demonstrated the role of CLA, the most active antioxidant component in fat milk, as an anti-atherogenic, anti-inflammatory, antioxidative, and anti-carcinogenic compound [21]. More generally, the antioxidant properties of the bioactive compounds from milk were preliminarily evaluated on in vitro models. Among these, Caco-2 cells (human colon adenocarcinoma cells) were the most used model due to their ability to mimic the intestinal barrier, thus allowing to study metabolites’ adsorption and distribution to distant organs [22].

Since bovine milk is very rich in long-chain SFAs, it is commonly believed that its intake should be minimized to reduce the risk of cardiovascular diseases. However, this mantra needs to be revised because the relationship between the intake of specific SFAs and the corresponding physiological effect is conflicting; in fact, some SFAs might also exert important biological functions [23]. Recent evidence reports that myristic acid, mainly responsible for the accumulation of fat in the body [24], has a positive effect on the cardiovascular system if the balance between the SFA and simple dietary carbohydrates in the diet is respected [25]. Furthermore, other evidence indicates that it is directly involved in mechanisms that control important metabolic processes in the human body through the activation of specific proteins [25,26]. Due to the numerous contradictory findings on the role played by the fat fraction, it is difficult to evaluate the risk–benefit ratio deriving from the consumption of milk. However, despite controversial opinions, bovine milk is preferred by Western populations for its pleasant taste, although some people choose to consume milk from other animals, such as goat, sheep, and donkey, because of food intolerances or allergies to some of its components. Among them, goat milk is considered healthier than bovine milk because of a better composition in MUFAs, PUFAs, and medium-chain TAGs. It is distinguished from bovine milk by the smaller size of its MFGs and the lower level of the cholesterol [27]. Because of its balanced FA profile, it could help to prevent heart diseases. Goat milk has also been reported to exert antioxidant and anti-inflammatory effects in the body [28].

As an alternative to animal milks, a growing number of consumers opt for plant-based milk substitutes, such as soy, rice, corn, nuts, or almonds, that do not contain lactose and dairy proteins [29]. Vegetable milks contain FAs different from those of animals; MUFAs and PUFAs are more represented than saturated ones [2]. Vegetable milk substitutes have been reported to have positive health effects because of the high antioxidant activity and the FA profile, which could reduce the risk of cardiovascular diseases, cancer, atherosclerosis, and diabetes [30]. Soy milk, the water extract of soybean, is enriched in UFAs, while containing only a small amount of SFAs, and it lacks cholesterol or lactose. Therefore, it is considered as a suitable substitute for bovine milk and an ideal nutritional supplement for the lactose-intolerant population [31].

From a technical point of view, given the different compositions of the emulsions, the protocols for extracting lipids from milks must consider the differences in solubility and volatility of the different carbon chains of the FAs and remove or cancel the water that may be present in the sample. So far, the most common approach to quantify FAs is gas chromatography (GC) coupled to a flame ionization detector (FID) or mass spectrometer (MS) after fat transesterification [1]. Solvent extraction is widely used to separate fats from the sample [1,32,33,34,35,36,37]. In this regard, solvent mixtures, such as chloroform/methanol [32,33], hexane/diethyl ether [34], or diethyl ether/heptane [35], have been used in different proportions to obtain solvents with different degrees of polarity. Other solvent mixture used are those based on dichloromethane [36], *n*-hexane, or petroleum ether [37,38]. Although the protocols for extraction and analysis are well established, all these methods are time-consuming, laborious, expensive, and impractical for processing many samples. Furthermore, various classes of lipids are not recovered in the same way, due to the natural emulsion of the oil in the milk water and the size of MFGs (between 0.1 and 20 µm) [35]. A rapid separation of milk lipids can be also achieved without the use of solvents by an established protocol based on multiple centrifugations [39].

In this work, four types of commercial milks, two semi-skimmed animal milks (bovine and goat milk) and two vegetable ones (soy and rice milk), were selected for studying the substantial differences in their lipid fractions. Two different protocols, a nonsolvent method and a solvent extraction protocol, were used to recover the total and the free lipid fractions from each tested milk, respectively. The two different lipid fractions obtained by each milk, along with the raw milks, were tested on a Caco-2 cell line to examine how the viability of the intestinal cell line is affected by dietary fats, after a lipopolysaccharide (LPS) stimulation. Furthermore, the antioxidant activity of the tested milks and their lipid fractions were also evaluated by chemical and biological assays. The results obtained from this study support the findings reevaluating the health effect of animal milks compared to vegetable ones in an intestinal cellular model.

## 2. Results

### 2.1. Comparison of Two Different Protocols for the Recovery of Total and Free Lipid Fractions from Commercial Animal and Vegetables Milks

To investigate the health effect of free fatty acids (FFAs) in milks, it is important to consider their variation according to the extraction protocols. In this study, we compared the amount of fat recovered in commercial animal (bovine and goat) and vegetable (soy and rice) milks by using two different protocols: a nonsolvent method and a solvent extraction method. More specifically, a lipid separation protocol based on sequential centrifugations without the use of a solvent [39] allowed us to obtain the total milk fat from the selected samples, while ethyl acetate was used as a solvent to extract the free lipid fraction from the same samples.

**Figure 1:**
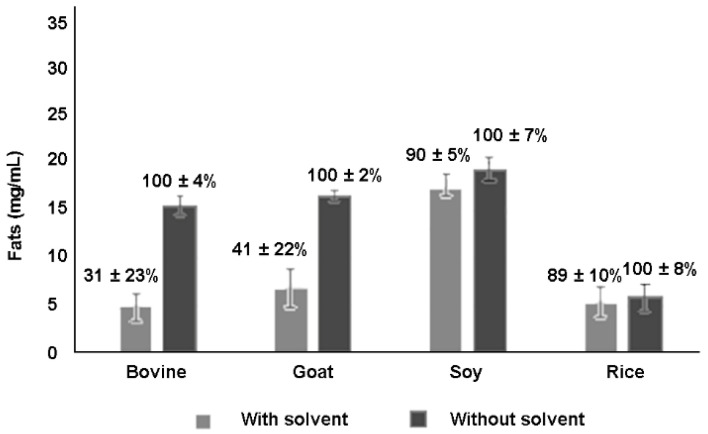
Amount of the free (gray) and total milk fat (black) extracted from 1 mL of commercial animal (bovine and goat) and vegetable (soy and rice) milks, and their relative percentage as recovered by the ethyl acetate extraction protocol and by the nonsolvent method, respectively, relatively to the quantity indicated on the milk label (number of replicates, *n* = 6).

Figure 1 shows the amount of total milk fat and the corresponding free lipid fraction recovered from 1 mL of each milk without or with the use of solvent, respectively.

Centrifugation was found to be generally very efficient in recovering the total fat from milks, as no difference was found for all the tested milks. On the contrary, the amount of the free lipid fraction recovered by ethyl acetate extraction was lower in animal than in vegetable milks, in which a percentage of recovery ≥89% was reached.

### 2.2. Free Fatty Acids Profile of Commercial Animal and Vegetables Milks Obtained by GC-MS Analysis

**Figure 2:**
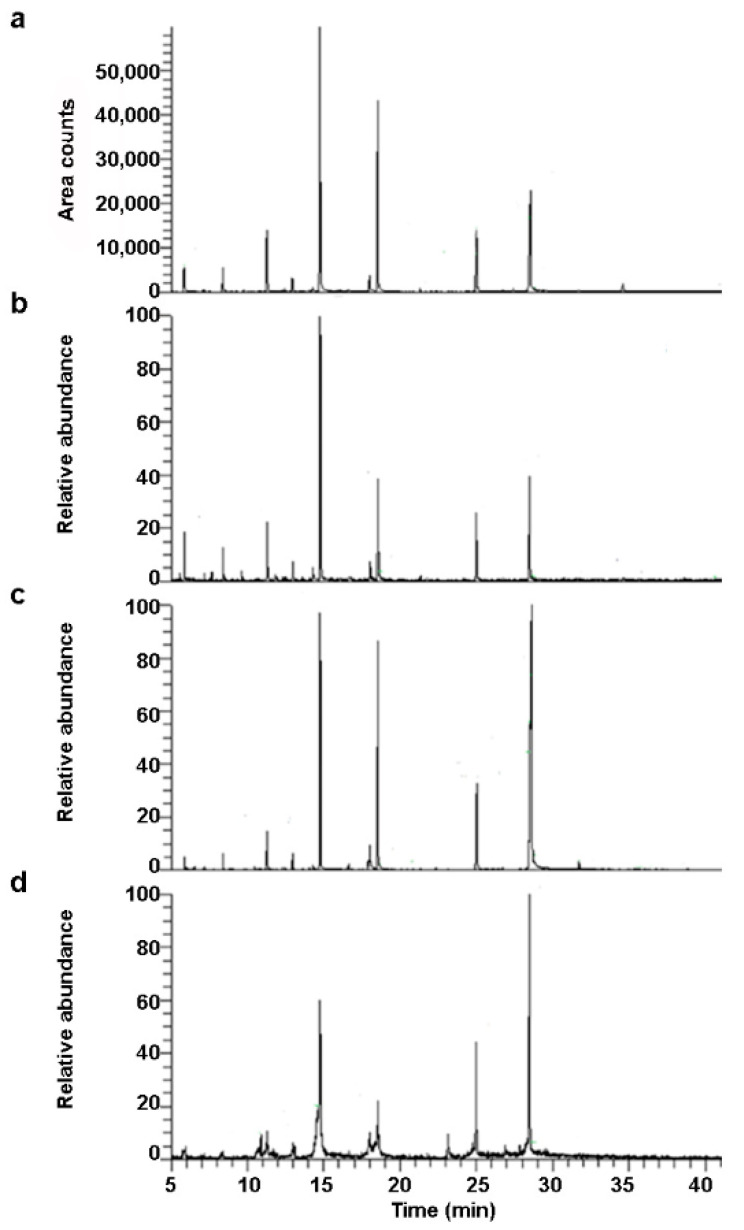
GC-MS XICs (ion 117 *m/z*, time window 5–40 min) related to the analysis of milk samples extracted with solvent from bovine milk (**a**), goat milk (**b**), soy milk (**c**), and rice milk (**d**).

The GC-MS analysis allowed us to obtain different lipid profiles for the milk samples, highlighting important differences among the samples. The FA profiles were produced replicating the measures three times for each type of milk. The spectra of the trimethylsilyl (TMS) derivatives of FFAs are generally easy to recognize, as they are characterized by two intense ions at 73 and 117 *m/z*, which correspond to the [(CH_3_)_3_Si]^+^ and [(CH_3_)_3_SiCO_2_]^+^ fragments, respectively. The molecular ions have a low intensity, while very intense M-15 fragments are always present, deriving from the loss of a methyl unit from the trimethylsilyl group, and allowing us to identify FAs with the same number of C atoms, but a different degree of unsaturation. Figure 2 shows the GC-MS extracted ion chromatograms (XICs) (ion 117 *m/z*, time window 5–40 min) related to the analysis of milk samples extracted with a solvent from bovine milk (a), goat milk (b), soy milk (c), and rice milk (d), respectively.

Each milk showed a specific chromatographic profile. The lipids identified using the NIST mass spectral library are listed in Table 1.

As expected, the identified lipids were mainly TMS esters of FFAs and monoacylglycerols (MAGs) (Table 1). Trimethylsilyl ether cholesterol was only detected in animal milks, of which bovine milk was ten times richer than goat milk. The samples were poor in short-chain FAs (C4-10), probably because of the drying procedure required by the GC analysis. Except for the loss of volatile FAs, the two protocols showed that the major FAs in the milks were equally represented.

**Figure 3:**
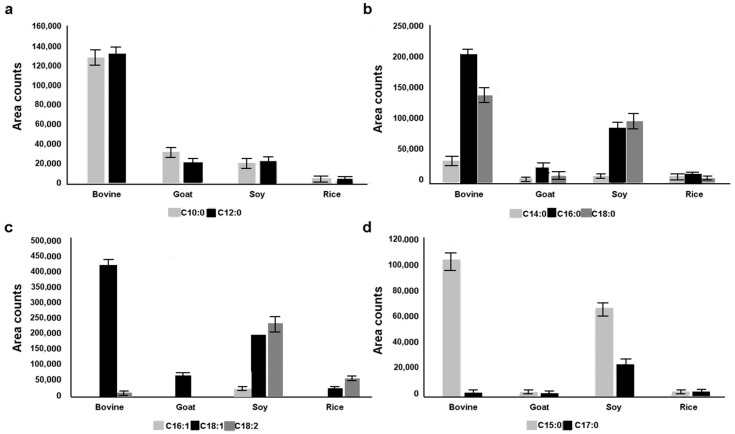
GC-MS area counts of FFAs in the solvent-extracted TMS derivates from the commercial animal and vegetable tested milks. Amount of (**a**) medium-chain FAs (capric and lauric acid, respectively), (**b**) SFAs (myristic, palmitic, and stearic acid, respectively), (**c**) UFAs (palmitoleic, oleic, and linoleic acid, respectively), (**d**) odd-chain fatty acids (OCFAs) (pentadecanoic and margaric acid, respectively) in bovine, goat, soy, and rice milks.

In agreement with previous reports [27,40,41], bovine milk was characterized by a higher content of medium-chain SFAs (capric and lauric acid, C10:0 and C12:0, respectively) compared to goat and vegetable milks (Figure 3a).

Capric and lauric acid were under-represented in rice milk compared to the other tested milks (Figure 3a). As shown in Figure 3b, the most abundant FFAs were long-chain SFA, i.e., myristic (C14:0), palmitic (C16:0), and stearic acid (C18:0). Palmitic acid was the dominant peak in all the analyzed samples except for soy milk, in which stearic acid was the most concentrated. Moreover, although to a lesser extent, myristic acid was more abundant in bovine milk. Some UFAs with known beneficial effects for human health, such as the ω-7 (palmitoleic acid, C16:1) and ω-9 (oleic acid, C18:1) MUFAs and the ω-6 (linoleic acid, C18:2) PUFA, were also identified in the extracted derivatives of the tested milks. However, palmitoleic acid was only found in the soy milk, although at a lower concentration compared to the other UFAs. Bovine milk contained higher levels of oleic acid compared to the other milks, while linoleic acid was more abundant in vegetable milks, especially in soy milk (Figure 3c). Odd-chain fatty acids (OCFA), such as pentadecanoic (C15:0) and margaric acid (C17:0), were also detected in bovine and soy milk extracts (Figure 3d), but it was not possible to discriminate between the different isoforms (iso and anteiso). Although pentadecanoic acid was more abundant in bovine milk than in soy milk, margaric acid was only detected in soy milk. The presence of these FAs is associated with the gut microbiome of ruminants, supporting their antioxidant activity [42,43].

### 2.3. Trolox Equivalent Antioxidant Capacity (TEAC) of Commercial Animal and Vegetables Milks

To investigate the antioxidant capacity of the animal and vegetable raw milks and their total and free lipid fractions, in terms of their radical scavenging activity, the Trolox equivalent antioxidant capacity (TEAC) was measured, by observing the samples’ interaction with 1,1-diphenyl-2-picrylhydrazyl (DPPH), a violet-colored stable radical that absorbs strongly at 517 nm. In general, the TEAC of each raw milk was much higher, at least two orders of magnitude, than that of the corresponding lipid fraction, recovered by both separation methods, indicating that the greater antioxidant activity is due to the non-lipid fraction (Table 2).

Among the raw milks, the rice one seemed to have the lowest antioxidant capacity. By comparing the TEAC values obtained by the lipid fractions from 1 mL of milks with and without the use of solvent, a higher antioxidant effect was noted for bovine and rice milks, comparable to goat milk, and a lower effect for soy in the lipid fraction obtained with solvent than in the one without solvent. Considering the TEAC values for the g of extracted fats, the fraction obtained with solvent had a higher antioxidant activity than those obtained without solvent.

### 2.4. Modulation of Caco-2 Cell Viability by Animal and Vegetable Milks as Assessed by an MTT Assay

**Figure 4:**
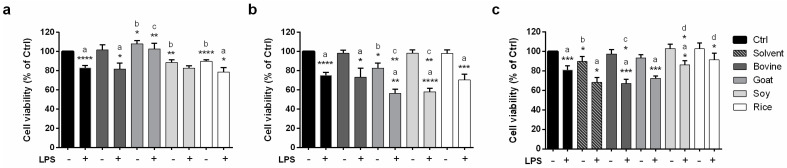
Effect of raw milks and their total and free lipid fractions on Caco-2 cell viability as assessed by an MTT assay. The bars represent the calculated mean value of cell viability percentages vs. Ctrl ± SEM (*n* = 3) relatively to Caco-2 cells treated with raw milks (**a**) and their total lipid and free lipid fractions (25 µg/mL, (**b**,**c**), respectively) at basal conditions and 48 h after LPS stimulation. Ctrl: raw milks: only cells; total lipid and free lipid fractions from crude milks: DMSO. a: LPS-stimulated vs. unstimulated samples; b: crude milks/milk extracts treated samples vs. unstimulated control sample; c: crude milks/milk extracts treated samples + LPS vs. control sample + LPS; d: crude milks/milk extracts treated samples + LPS vs. solvent + LPS. * *p* < 0.05, ** *p* < 0.01, *** *p* < 0.001,**** *p* < 0.0001.

To study the biological effects of the tested animal and vegetable milks, we first analyzed their ability to modulate Caco-2 cells’ viability after an inflammatory stimulus. After preliminary tests to select the right dose and timing (data not shown), we treated Caco-2 cells with both animal and vegetable raw milks (Figure 4a), their total lipid fraction (Figure 4b), and their free lipid fraction (Figure 4c) at a dose of 25 µg/mL, at basal condition, and 48 h after LPS stimulation. As shown in Figure 4a, the reduction of the cell viability induced by LPS relatively to the unstimulated samples was prevented when Caco-2 cells were treated with goat and soy raw milks compared to untreated cells and to cells treated with bovine and rice raw milks (Figure 4a; statistical analysis: a). Moreover, vegetable raw milks lead to a slight reduction of the cell viability relative to unstimulated control cells at basal conditions (Figure 4a; statistical analysis: b). On the contrary, only goat milk showed a pro-proliferative effect both at basal conditions and 48 h after LPS stimulation compared to unstimulated and LPS-stimulated control cells (Figure 4a; statistical analysis: b and c).

As regards the effect of the total lipid fraction of commercial animal and vegetable milks on Caco-2 viability, only goat milk induced a mild reduction of the viability at basal conditions relatively to the unstimulated control sample (Figure 4b; statistical analysis: b). This effect is even more pronounced after LPS stimulation for all the tested milks compared to the corresponding unstimulated samples (Figure 4b; statistical analysis: a), especially with goat and soy milks, for which a significant reduction in cell viability relatively to the control LPS-stimulated sample was detected (Figure 4b; statistical analysis: c).

The free lipid fraction from both the animal milks and soy milk induced a reduction of Caco-2 cell viability only after LPS stimulation relatively to the corresponding unstimulated sample (Figure 4c; statistical analysis: a). This effect was also significant when compared to the control LPS-stimulated sample, but only in the case of the free lipid fraction from bovine milk (Figure 4c; statistical analysis: c). No reduction in the cell viability induced by the free lipid fraction from rice milk was detected. Moreover, the effect of the free lipid fraction from animal milks, but not from vegetable milks, seemed to be mediated by the solvent used in the extraction protocol (Figure 4c; statistical analysis: d), although the solvent per se only slightly reduced the cell viability at basal conditions relatively to the unstimulated control sample (Figure 4c; statistical analysis: b).

### 2.5. Antioxidant Potential of Animal and Vegetable Milks Tested on Caco-2 Cells by a DCF Assay

**Figure 5:**
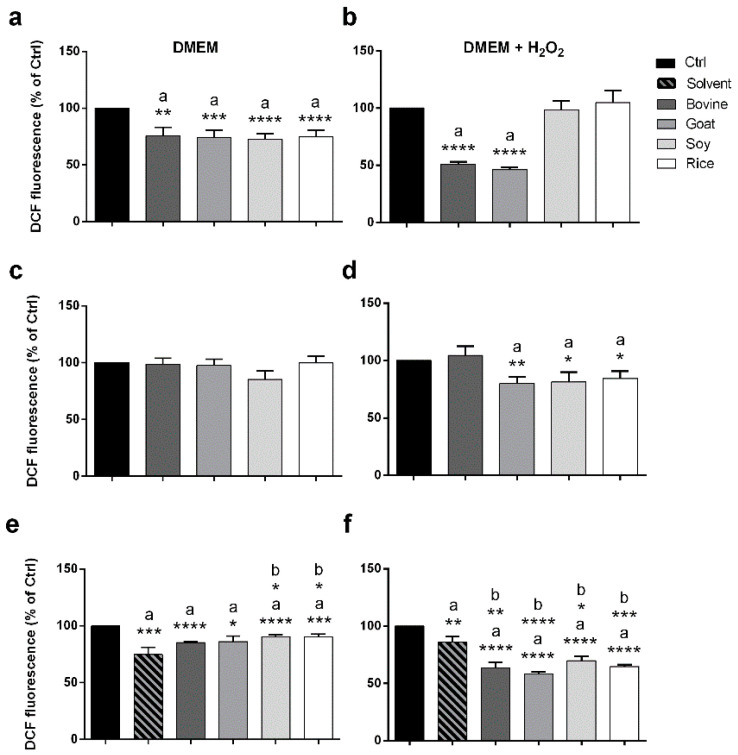
Antioxidant ability of raw milks and their total and free lipid fractions as assessed by a DCF assay. The bars represent the mean value of DCF fluorescence intensity relative to the Ctrl ± SEM (*n* = 3) of Caco-2 cells treated with raw milks (**a**,**b**) and their total and free lipid fractions (25 µg/mL, (**c**,**d**) and (**e**,**f**), respectively) before (**a**,**c**,**e**, respectively) and after (**b**,**d**,**f**, respectively) H_2_O_2_ stimulation. Ctrl: crude milks: only cells; total and free lipid fractions from crude milks: DMSO. *a*: sample vs. Ctrl; *b*: sample vs. solvent. * *p* < 0.05, ** *p* < 0.01, *** *p* < 0.001, **** *p* < 0.0001.

To study the antioxidant ability of the tested animal and vegetable milks, the modulation of reactive oxygen species (ROS) production in Caco-2 cells was tested by the dichlorofluorescein (DCF) assay. Animal and vegetable crude milks were both able to reduce the production of ROS at basal conditions compared to the control sample (Figure 5a; statistical analysis: a).

On the contrary, after H_2_O_2_ stimulation, this capacity was retained only by the animal milks, showing an even more significant reduction (49% and 54% for bovine and goat raw milks, respectively) (Figure 5b; statistical analysis: a). Furthermore, the total lipid fraction of both vegetable milks and goat milk showed a mild antioxidant ability only after H_2_O_2_ stimulation (Figure 5c,d; statistical analysis: a). Conversely, the free lipid fraction from both animal and vegetable milks slightly reduced the ROS production at basal conditions (Figure 5e; statistical analysis: a). This effect could be mediated by the solvent used in the extraction protocol for animal but not for vegetable milks (Figure 5e; statistical analysis: b). Moreover, the ROS reduction, that was more pronounced after H_2_O_2_ stimulation (Figure 5f; statistical analysis: a), was not solvent-dependent for all the tested samples (Figure 5f; statistical analysis: b).

## 3. Discussion

The possibility of having sensitive methods capable of detecting quantitative and qualitative differences between animal and vegetable milks could be of great help to better define their nutritional and health properties. In this study, four commercial samples of animal (bovine and goat) and vegetable (soy and rice) milks were investigated in terms of their total and free lipid fractions. The different lipid fractions were recovered using two different protocols. Specifically, the total lipid fraction of the four milks was obtained by sequential centrifugations (without adding any solvent) according to a procedure described by Feng et al. [39]. Conversely, ethyl acetate was used for the first time to extract the free lipid fraction not structurally linked to the milk fat matrix but associated with it through weak chemical-physical interactions. This solvent was selected among others due to its low cost, low toxicity, and affinity with carboxylic and dicarboxylic acids, alcohols, and carbohydrates [44]. As expected, the efficiency of the extraction was strongly related to the solubility of the lipids in the chosen solvent, and therefore to the length of their carbon chains. However, this is a common limit of solvent-based extraction methods.

From the acquired GC-MS chromatograms, it was possible to observe differences between the FA peak areas which seem to reflect their actual presence in the milks considered, except for the goat milk, for which a greater contribution of short-chain FAs (C < 10) was expected [45]. This discrepancy could be rationalized considering the higher volatility of short-chain FAs compared to other lipids and the limit of the technology used (which requires high operating temperatures). Considering the percentage of fat recovered by the two different procedures, the use of ethyl acetate as solvent produced a lower recovery in animal milks compared to the vegetable ones. Nevertheless, this discrepancy is probably due to intrinsic differences between the quantity and type of lipids that constitute them.

Medium-chain FAs were present especially in bovine milk, thus supporting some positive effects linked to this type of milk. Specifically, capric and lauric acids can reduce inflammation thanks to their ability to inhibit COX-I and COX-II [46]. Moreover, they have antiviral and antibacterial functions, as reported for *Helicobacter pylori* infection [47,48]. However, bovine milk also has higher levels of long-chain FAs, i.e., palmitic and stearic acid, although the latter is poorly absorbed by the gut, is not atherogenic, and does not increase serum cholesterol concentration [49].

Concerning the percentage of monounsaturated (C16:1, C18:1) and polyunsaturated (C18:2) FAs, it was possible to note that palmitoleic acid (C16:1) was only present in soy milk, while oleic acid (C18:1) was present in all kinds of milk, although with a much higher percentage in those of animal origin. Oleic acid seems to have a positive effect on human health due to its ability to reduce the concentration of plasma cholesterol, LDL-cholesterol, and TAGs [50], as well as to reduce intestinal inflammation and carcinogenesis [51]. On the contrary, linoleic acid (C18:2) was present only in vegetable milks, and showed undetectable levels in animal ones. This could partially explain the commonly beneficial role recognized for vegetable milks due to the antibacterial properties of linoleic acid, as also reported by the decreased invasiveness of *Listeria* monocytogenes in Caco-2 cell lines, along with its involvement in inflammatory pathways [52]. In fact, linoleic acid is required for the synthesis of eicosanoids and arachidonic acid [53].

OCFA were also generally detected, although it was not possible to differentiate between the different isoforms. As expected, bovine milk had the greatest amount of odd-branched-chain FAs (C15:0 and C17:0), due to their massive presence in the lipidic membranes of rumen bacteria [36,41]. The bacteria responsible for this are the rumen bacteria of the lipid membranes [42,43]. OCFA have antioxidant properties, acting as hydroxyl radical scavengers, and an increased dietary intake of OCFA was shown to be associated with a decrease in serum cholesterol [43].

Finally, the marked difference observed in cholesterol concentration in milks of animal origin could be due to a more prominent abundance of medium- and short-chain FAs in goat rather than in bovine milk, inhibiting cholesterol formation. The role of cholesterol in human health regulation is still controversial. Although a little contribution of dietary cholesterol to the regulation of plasma cholesterol levels has been reported, recent evidence also reported its positive role in the growth and development of infants [4].

Regarding the antioxidant capacity, TEAC values for raw milks differ among the four tested milks, underlying their peculiar lipid composition. When considering TEAC values for the g of fat for each milk, the free lipid fractions obtained by using the solvent-extraction method showed a higher antioxidant activity than those obtained by centrifugation, demonstrating a potentially better nutraceutical quality of the lipid content found through the former protocol. Importantly, although rice raw milk presented the lowest TEAC value, maybe reflecting its limited fat content, it still retained a good antioxidant ability in its lipid fraction.

The results from the chemically evaluated antioxidant ability were partially recapitulated by DCF assay on Caco-2 cells, for which similar papers are not reported in the literature. Specifically, bovine and goat raw milks, but not soy raw milk, were confirmed to have a higher antioxidant ability compared to rice raw milk in Caco-2 cells after H_2_O_2_ stimulation. A good correspondence for both animal and vegetable milks were found between the TEAC values obtained with solvent extraction and the ROS reduction on Caco-2 cells when stimulated with H_2_O_2_. Moreover, as for the chemical assay, a reduction of the antioxidant ability in the total lipid fraction relatively to the free lipid fraction was also found in the biological assay for all the tested milks, except for bovine milk. Although the percentage of recovery of fats from animal milks obtained by using the solvent-extraction method is generally low, they showed an antioxidant ability comparable to those obtained by vegetable milks, thus reevaluating the beneficial role of animal milk in terms of antioxidant potential. With the intent to identify the lipid molecules regulating the antioxidant potential of the tested samples, it is possible to speculate that both SFAs (palmitic, stearic, capric, and lauric acids—the latter two mainly for bovine milks) and UFAs (oleic and linoleic acids, the latter only for soy milk), as well as margaric and pentadecanoic acids (the latter only for bovine milk) might be involved in bovine and soy free lipid fractions. On the other hand, the FFAs identified in goat and rice milks contribute only minimally to their antioxidant activity, especially in rice milk. It is thus possible to suppose that other components in these milks, apart from the free lipid fraction, could contribute to their beneficial effect.

Finally, in this study the effects of commercial animal and vegetable milks on the modulation of cell viability after the LPS stimulation of human intestinal Caco-2 cells were examined. The Caco-2 cell line, deriving from human colon adenocarcinoma, has enterocyte-like characteristics and has been widely used as an in vitro model of absorption by intestinal epithelial cells [54,55,56]. LPS is the main component of the outer membrane of Gram-negative bacteria, playing a pivotal role in triggering the inflammatory response. Results indicated that goat raw milk has a pro-proliferative effect not only at basal conditions but also after an inflammatory stimulus, while soy raw milk was able to prevent the reduction in cell viability only after LPS stimulation. The total lipid fraction from goat milk negatively modulated Caco-2 viability both after LPS stimulation and at basal conditions, while the same fraction from soy milk reduced the cell viability only after LPS stimulation. The free lipid fraction from the tested milks could preserve the cell viability only in the case of rice milk, emphasizing the importance of the quality rather than the quantity of the lipid profile relatively to the beneficial health effects. Conversely, the bovine free lipid fraction significantly reduced Caco-2 viability also relatively to Ctrl LPS-stimulated cells. Considering the specific free lipid profile of bovine milk described before, including SFAs with beneficial properties, these data could be explained using a lipid extract instead of commercial single molecules [57]. However, most of the articles studying the beneficial effects of lipid components on in vitro models, investigated other aspect of the inflammation, also focusing on the molecular and metabolic pathways activated [58,59]. In fact, it is commonly known that the bioactive compounds of food have a crucial role in this context, thus supporting their ability to prevent chronic diseases [60,61].

## 4. Materials and Methods

### 4.1. Collection and Preparation of Milk Lipid Samples

Four types of milks, two semi-skimmed of animal origin milks (bovine and goat milk) and two vegetables ones (soy and rice milk), were purchased from local stores. The total fat content shown on the labels was 1.55, 1.60, 1.80, and 0.60% (*w*/*v*) for bovine, got, soy, and rice milk, respectively. The total lipid fraction was extracted by the milks by centrifugation, as already reported by Feng et al. [39]. Briefly, 10 mL of fresh milk in a 15 mL conical plastic tube was centrifuged at 17,800× *g* for 30 min at 4 °C. An aliquot (∼1.0 g) of the fat-cake layer was transferred to a 1.5 mL microtube and left at room temperature (∼20 °C) for approximately 20 min until the fat cake melted. This was then centrifuged at 19,300× *g* for 20 min at room temperature by a microcentrifuge (Z 216 MK, Hermle Labortechnik GmbH, Wehingen, Germany). After centrifugation, the top layer was recovered and dissolved in dimethyl sulfoxide (Sigma-Aldrich, St. Louis, MO, USA). The separation protocols were both replicated six times for each type of milk. Instead, the free lipid fraction was separated by extraction with a solvent. More specifically, 4 mL of ethyl acetate (Sigma-Aldrich, MI, Italy) was added to a milk aliquot (20 mg of fat) containing 1:10 (*v*/*v*) 1 M sodium acetate buffer, pH 5. The tube was shaken vigorously for 15 min at room temperature and left vertically for the time required for the separation of the two phases (to improve the separation, a short spin at 221× *g* was applied for 30 s). The extractions were repeated four times on each sample. The recovered upper phases were combined and dried by means of a CHRIST-vacuum concentrator (RVC 2–18, Osterode am Harz, Germany) set at 60 °C. The dry residues were dissolved in dimethyl sulfoxide (Sigma-Aldrich, St. Louis, MO, USA) to perform the biological tests.

### 4.2. Total Antioxidant Capacity (TAC) Assay

The assay was performed according to a slightly modified literature protocol [62]. An aliquot (0.05 mL) of each sample in dimethyl sulfoxide (i.e., either raw milk (diluted 1:10, *v*/*v*) or the lipid fractions obtained by the solvent or the nonsolvent method at levels of 50 and 200 mg/mL, respectively)) was transferred to a test tube containing 0.95 mL of an ethanolic solution of 0.08 mM DPPH (2,2-diphenyl-1-picrylhydrazyl, Sigma-Aldrich, St. Louis, MO, USA) (S) or pure ethanol (B). The tubes were kept in the dark at room temperature for one hour. The absorbance was read at 515 nm against a 5% dimethyl sulfoxide ethanolic solution in a spectrophotometer (Shimadzu UV 1650 PC, Milano, Italy). The radical scavenging activity (RSA) was expressed as the percentage of the DPPH consumed, which was calculated using the following formula:

% of RSA = 100 − [(Abs_S_ − Abs_B_)/Abs_DPPH_] × 100

where Abs_S_ is the absorbance of the sample with DPPH, Abs_B_ is the absorbance of the sample without DPPH, and Abs_DPPH_ is the absorbance of the DPPH solution.

The % of RSA was used for calculating the total antioxidant capacity (TAC), which was expressed as the Trolox equivalent antioxidant capacity (TEAC) (mmol of Trolox/1 mL of milk).

### 4.3. Determination of Milk Lipid Fraction by a GC-MS Analysis

The FFAs separated by solvent extraction were characterized using a gas chromatograph coupled with a mass spectrometer (GC-MS) after chemical derivatization. More specifically, an aliquot (5 µL) of the organic phase of the animal or vegetable milk was dried by means of a CRHIST AVC 2–18 set at 60 °C into a 250 µL glass vial (Supelco, MI, Italy). A 4:1 (*v*/*v*) *N*,*O*-bis(trimethylsilyl)trifluoroacetamide (BSTFA, Sigma-Aldrich):pyrimidine (Sigma-Aldrich) freshly-prepared mixture (25 µL) was added to the dry residue. The vial was closed with a perforated cap and incubated in a Thermo Shaker at 70 °C for 10 min, under gentle stirring. Finally, the reaction mixture was allowed to cool for a few minutes at room temperature to be promptly injected (1 µL) into the inlet port of the GC maintained at 250 °C.

#### Apparatus

A Finnigan Trace GC Ultra (Thermo Fisher Scientific, Waltham, MA, USA) gas chromatograph equipped with an ion-trap mass spectrometer (Polaris Q, Thermo Scientific, MI, Italy) was used. A chromatographic separation was performed with a TRACE TR-5 SPB-5 fused-silica capillary column (30 m × 0.25 mm i.d., 0.25 mm film thickness) (Thermo Fisher Scientific, Waltham, MA, USA). Helium was the carrier gas, with a constant flow rate of 1.0 mL/min. The column temperature program was set as follows: 130 °C for 3 min, a 4 °C/min increase until 280 °C, and then 15 min at this temperature.

The mass spectrometer was operated in the electron impact positive (EI+) ionization mode with the source set at 220 °C. The electron energy was 70 eV, and the filament current 150 µA. Mass spectra were acquired in the 50–600 *m/z* range (0.55 scans/s). Analytes were detected using extracted-ion chromatograms, extracting the 117 *m/z* ion.

Chromatographic peak identification was carried out by a comparison of the volatile sample mass spectra with spectra in the National Institute of Standards and Technology (NIST) library (Available online: http://webbook.nist.gov/chemistry, accessed on 23 August 2021).

### 4.4. Cell Culture

Human colorectal adenocarcinoma cells (Caco-2) were purchased from the American Type Culture Collection (ATCC, Manassas, VA, USA) and maintained at 37 °C in a humidified atmosphere with 95% air and 5% CO_2_, and they were periodically screened for contamination. Cells were grown in Dulbecco’s modified Eagle’s medium (DMEM)—high glucose (Sigma-Aldrich, St. Louis, MO, USA) supplemented with 10% Fetal Bovine Serum (FBS, Sigma-Aldrich, St. Louis, MO, USA), 1% L-glutamine (Sigma-Aldrich, St. Louis, MO, USA), 100 U/mL penicillin/streptomycin (Sigma-Aldrich, St. Louis, MO, USA), 1% Non-Essential Amino Acids (NEAA) (Sigma-Aldrich, St. Louis, MO, USA), and 1% sodium pyruvate (Sigma-Aldrich, St. Louis, MO, USA).

### 4.5. Cell Viability Assay

Caco-2 cell viability was assessed by using a conventional 3-(4,5-dimethylthiazol-2-yl)-2,5-diphenyltetrazolium bromide (MTT) assay as previously reported [63,64]. Briefly, viable cells (2.5 × 10^4^ cells/well) were seeded and grown for 24 h in a complete medium into a sterile 96-well cell culture cluster (Corning, Sigma-Aldrich, St. Louis, MO, USA) and maintained at 37 °C in a humidified atmosphere with 5% CO_2_. Subsequently, the cells were exposed to crude milks and their extracts with the total and the free lipid fractions at the concentrations of 25 µg/mL and stimulated with 1 µg/mL of lipopolysaccharide (LPS, Sigma-Aldrich, St. Louis, MO, USA) for 48 h. For raw milks, the same volume used for their total lipid fraction was added. At the end of the LPS stimulation, the culture medium was replaced by a solution of 0.5 mg/mL MTT (Sigma-Aldrich, St. Louis, MO, USA) in PBS. After 2 h of incubation at 37 °C in 5% CO_2_, the supernatant was carefully removed from each well, and the formazan crystals were dissolved in DMSO (Sigma-Aldrich, St. Louis, MO, USA). The absorbance values were measured at 570 nm using a TECAN M1000 pro using DMSO medium as blank solution. The cell viability was calculated according to the following formula: % cell viability = [optical density (OD) of tested compound/medium OD of control cells] × 100.

### 4.6. Dichlorofluorescein Assay

The generation of ROS was monitored using an oxidation-sensitive fluorescent probe, 2′-7′-dichlorodihydrofluorescein diacetate (DCFH-DA, D6665; Sigma-Aldrich, St. Louis, MO), by slightly modifying the procedure reported by Wang and James [65]. Briefly, viable Caco-2 cells (5 × 10^4^ cells/well) were plated into a sterile black Culture Plate™ 96F wells (Costar, Sigma-Aldrich, St. Louis, MO, USA) the day before the experiments. The cells were treated with 25 µg/mL of crude milks, and their extracts with the total and the free lipid fractions. For raw milks, the same volume used for their total lipid fraction was added. After 1 h of treatment, the cells were washed with 1X PBS (Sigma-Aldrich, St. Louis, MO, USA) and incubated with 25 µM DCFH-DA in DMEM for 30 min at 37 °C in 5% CO_2_ in the dark. The medium was removed, and the cells were washed with 1X PBS and incubated with final 100 µM H_2_O_2_ in DMEM at 37 °C in 5% CO_2_ for an additional 30 min. The samples were excited at 485 nm, and the fluorescence was measured at 530 nm by TECAN M1000 PRO (Thermo Fisher Scientific, Waltham, MA, USA).

### 4.7. Statistical Analysis

The statistical analysis was performed using the Graphpad Prism statistical software (release 6.01) for Windows XP. All biological data were expressed as means ± SEM obtained from at least three independent experiments. The statistical significance was evaluated by two-tailed Student’s *t*-test. Results were considered statistically significant at *p* < 0.05.

## 5. Conclusions

The state-of-art of the relationship between milk’s lipid profile and human health is still intricate and somewhat controversial, especially in the case of SFA. The present study demonstrated, for the first time, the importance of the lipid profile in the modulation of the antioxidant ability and cellular viability in response to an inflammatory stimulus in Caco-2 cells. These beneficial effects could rely on the specific lipid composition of the commercial animal and/or vegetable tested milks, as well as on the extracted total or free lipid fractions, depending on the protocol used. Importantly, in line with part of the literature, this paper reevaluates animal milks relatively to vegetable ones thanks to their potential beneficial health effects. However, more *in vitro* and *in vivo* studies are needed to dissect the metabolic pathways supporting these biological effects, trying to identify the specific metabolites involved in these cellular processes.

## Figures and Tables

**Table 1:** List of lipids identified by GC-MS in the TMS-derivatized milk samples.

#	RT * (min)	Compound	Formula	Common Name and Double Bond Position
1	5.87	Heptanoic acid, tert-butyldimethylsilanyl ester	C_13_H_28_O_2_Si	(FFA) C10:0 *Capric acid*
2	8.40	Dodecanoic acid, trimethylsilyl ester	C_15_H_32_O_2_Si	(FFA) C12:0 *Lauric acid*
3	11.28	Tetradecanoic acid, trimethylsilyl ester	C_17_H_36_O_2_Si	(FFA) C14:0 *Myristic acid*
4	12.95	Tetradecanoic acid, 6-methyl, trimethylsilyl ester	C_18_H_38_O_2_Si	(FFA) C15:0 *Pentadecanoic acid*
5	14.29	Hexadecanoic acid, trimethylsilyl ester	C_19_H_38_O_2_Si	(FFA) C16:1 *Sapienic acid (6)* *Palmitoleic acid (9)*
6	14.77	Hexadecanoic acid, trimethylsilyl ester	C_19_H_40_O_2_Si	(FFA) C16:0 *Palmitic acid*
7	16.63	Heptadecanoic acid, trimethylsilyl ester	C_20_H_42_O_2_Si	(FFA) C17:0 *Margaric acid*
8	17.92	17-Octadecynoic acid, trimethylsilyl ester	C_21_H_40_O_2_Si	(FFA) C18:2 *Linoleic acid (9,12)* *Rumenic acid (cis-9, trans-11)*
9	18.01	Oleic acid, trimethylsilyl ester	C_21_H_42_O_2_Si	(FFA) C18:1 *Oleic acid (9)* *Eleaidinic acid (trans-9)* *Vaccenic acid (trans-11)* *Asclepic acid (11)* *Petroselaidic acid (trans-6)*
10	18.56	Octadecanoic acid, trimethylsilyl ester	C_21_H_44_O_2_Si	(FFA) C18:0 *Stearic acid*
11	21.31	Myristic acid, 2-(trimethylsiloxy)-1-[(trimethylsiloxy)methyl]ethyl ester	C_23_H_50_O_4_Si_2_	(MAG) C14:0
12	23.16	Pentadecanoic acid, 1,3-bis-(OTMS) propyl ester (β-glyceryl pentadecanoate)	C_24_H_52_O_4_Si_2_	(MAG) C15:0
13	24.98	Hexadecanoic acid, 2,3-bis[(trimethylsilyl)oxy]propyl ester	C_25_H_54_O_4_Si_2_	(MAG) C16:0
14	26.70	Heptadecanoic acid, glycerine-(1)-monoester, bis-*O*-trimethylsilyl	C_26_H_56_O_4_Si_2_	(MAG) C17:0
15	28.58	Octadecanoic acid, 2,3-bis[(trimethylsilyl)oxy]propyl ester	C_27_H_58_O_4_Si_2_	(MAG) C18:0
16	31.70	Eicosanoic acid, 2,3-bis[(trimethylsilyl)oxy]propyl ester	C_29_H_62_O_4_Si_2_	(MAG) C20:0
17	34.57	Cholesterol trimethylsilyl ether	C_30_H_54_OSi	*Cholesterol*

* RT: retention time.

**Table 2:** Average TEAC values obtained by the raw milks and by the corresponding total and free lipid fractions obtained with the nonsolvent method (*n* = 3) and with solvent extraction (*n* = 3), respectively.

Sample	Milk (*n* = 3)	Nonsolvent Method Total Lipid Fraction (*n* = 3)		Solvent Extraction Free Lipid Fraction (*n* = 3)	
	(TEAC/mL_milk_)	(TEAC/mL_milk_)	(TEAC/g_extr.fats_)	(TEAC/mL_milk_)	(TEAC/g_totalfat_)
Bovine	48.04 ± 11.04	0.26 ± 0.10	16.66 ± 0.02	0.35 ± 0.02	72.91 ± 0.02
Goat	40.16 ± 5.96	0.28 ± 0.06	16.88 ± 0.04	0.28 ± 0.07	41.18 ± 0.07
Soy	40.25 ± 5.06	0.30 ± 0.08	16.11 ± 0.09	0.24 ± 0.11	38.26 ± 0.09
Rice	20.11 ± 3.06	0.21 ± 0.05	35.00 ± 0.06	0.25 ± 0.08	47.17 ± 0.04

## Data Availability

Not applicable.

## Sample Availability

Samples under study are from commercial sources.
